# Book Reviews

**Published:** 1916-05

**Authors:** 


### BOOK REVIEWS

**Books mentioned may be inspected at and ordered through this office. So far as possible, books received in any month will be reviewed in the issue of the second month following.**

---

**The American Year Book of Anesthesia and Analgesia.** Under the editorship of F. H. Mc Mechan, A. M., M. D., will shortly be issued by the Surgery Publishing Co. of New York.

This will comprise 352 pages, 8 1-4 x 11, profusely illustrated, price $4. The various topics reviewing the advances in 1915, will be treated by a corps of able writers.

---

**Digest of Comments on the U. S. P. & N. F.** For 1914. by Martin I. Willbert. Gov. Printing Office, Wash. U. S. P. H. S., Hygienic Laboratory, Bulletin No. 105. 516 pages, paper cover, 50 cents.

---

**Publications of the Buffalo Historical Society.** Vol. 19, 1915. Edited by Frank H. Severance, Secretary, published by the Society.

This includes a biographic sketch of the late J. N. Larned by John B. Olmstead, extracts from and a bibliography of his writings; a tribute to the late Henry A. Richmond by Henry R. Howland; articles on the early newspapers of Buffalo by J. C. Brayman, Joseph Stringham and Frederick J. Shepard and a bibliography of the Periodical Press of Buffalo by Frank H. Severance. The last mentions this Journal, the Medical Press of Western N. Y. and Das Medicinisch Chirurgisches Correspondenzblatt, the last two discontinued, as the medical publications of Buffalo.

---

**Gould's Practitioner's Medical Dictionary.** Third edition, revised and edited by J. E. Scott, M. A., B. C. L., M. D. of N. Y. xx plus 962 pages, flexible cloth, round corners, marbled edges, $2.75.

Nearly 71,000 terms are defined and pronounced, including modern additions to the medical vocabulary. 400,000 copies have already been sold of Gould's medical dictionaries, averaging more than one copy apiece for every physician of English-speaking countries. This fact and the well-known skill of Dr. George M. Gould and the reviser in compilation of this sort, are the only evidence necesary as to the high order of the work.

---

**The Medical Clinics of Chicago.** W. B. Saunders Co., Phildelphia, Bi-monthly, $8. Mch. 1916, Vol. 1, No. 5. 220 pages.

Jas. T. Case's clinic on Roentgenologic Aspects of Intestinal Stasis is the most striking article of this issue though the others are of great practical value.

---

**Transactions of the American Surgical Assn.** Vol. 33, 1915, edited by Dr. John F. Binnie, Recorder, Kansas City, Mo. 825 pages, illustrated.

This contains a large number of interesting monographs, covering various phases of surgery and borderline branches. The review of the literature on fractures and the report of a committee for collective investigation of this subject, deserve special attention.

---

**Hand Book.** of General Orders, Instructions and Rules, for Field Employes of the Bureau of Medical Inspection. Issued by the Dept. of Health, Chicago.

This is a small pamphlet of 182 pages, not only useful as a guide for those for whom it is especially intended, but serving as a model for other cities. It might be used to advantage by those interested in preparing themselves for service of this nature.

---

**Social Travesties.** And what they cost, by Dr. D. T. Atkinson, Dallas, Texas, published by the Vail-Ballou Co., N. Y. 152 pages, $1.00.

This book is intended particularly for lay instruction and deals with the venereal peril, and allied social topics, advocates early instruction on these matters and the enfranchisement of women.

---

**Progressive Medicine.** Edited by H. A. Hare and L. F. Appleman, Philadelphia, published by Lea & Febiger. quarterly, $6.00. Vol. 19, No. 1, Mch. 1916.

This volume deals with surgery of the head and neck and the thorax, infectious diseases, including acute rheumatism, croupous pneumonia and influenza, diseases of children, rhinology, laryngology and otology. The respective contributors are: Floyd M. Crandall, Chas. H. Frazier, Geo. P. Mueller, John Rurah, Truman L. Saunders, Geo. P. Wood.

---

**Transactions of the Am. Assn. of G. U. Surgeons.** Vol. 10, 1915, published for the association by Frederick H Hitchcock, 105 W. 40, N. Y. (Sec. Richard F. O'Neil, Boston). 339 pages, illustrated.

This contains a large number of articles on various branches of the specialty.

